# “It is more isolating to patients if you aren’t familiar with the resources”: a pilot test of a clinician sensitivity training on eating disorders in pregnancy

**DOI:** 10.1186/s12909-023-04894-x

**Published:** 2023-12-06

**Authors:** Zoya A. Khan, Christa L. Lilly, Caterina DeFazio, Elizabeth A. Claydon

**Affiliations:** 1 West Virginia University School of Medicine, Morgantown, WV USA; 2 Department of Biostatistics, West Virginia University School of Public Health, Morgantown, WV USA; 3 Department of Social and Behavioral Sciences, West Virginia University School of Public Health, Morgantown, WV USA

**Keywords:** Eating disorders, Pregnancy outcomes, Postpartum, Clinician education, Medical education, Continuing education

## Abstract

It is more isolating to patients if you aren’t familiar with the resources: A pilot test of a clinician sensitivity training on eating disorders in pregnancy.

**(**IRB Number: 1909705198).

**Background** Pregnant women with a history of eating disorders (EDs) or active EDs have greater maternal and child health complications. They are also unlikely to disclose their history with an ED to their clinician, few of which are confident in their knowledge to provide appropriate care for patients who present with EDs. This study’s goal was to evaluate changes to knowledge, behavior, and attitudes for health professionals who were part of a sensitivity training (to provide information of and awareness, address potential clinician biases, and offer strategies for more patient-centered care with de-stigmatizing language) about eating disorders and pregnancy compared with those who received a reference document.

**Methods** Our pilot study compared responses of health professionals before and after this sensitivity training (*N* = 54) with a group who were provided a clinician reference document about the same topic (*N* = 61).

**Results** Mann–Whitney Wilcoxon tests showed significant differences between the sensitivity training and reference document groups, with the sensitivity training resulting in increases to participants’ perception of ED’s relevance to overall treatment (*p* = 0.018), comfort in providing resources (*p* < 0.0001), frequency of ability to introduce strategies (*p* = 0.001), and interest in additional strategies/recommendations in treating patients with eating disorders (*p* = 0.009). Thematic analysis of the open-ended responses indicated four major themes: *Resources and support, Treatment, Additional training,* and *Clinician Strategies*.

**Discussion **Results indicated that the sensitivity training improved training outcomes compared to the reference document group. Qualitative responses from both groups indicated four themes that can help inform ED-centered care. This study provides context for future directions for continuing education courses as well as clinical training recommendations for treating pregnant patients with EDs.

**Supplementary Information:**

The online version contains supplementary material available at 10.1186/s12909-023-04894-x.

## Background

Eating disorders (EDs) are defined as abnormal eating patterns that adversely impact one’s physical and mental wellbeing and can affect all genders, ages, races, ethnicities, body shapes and weights, sexual orientations, and socioeconomic statuses [1–3]. EDs are more prevalent among women and can affect women during critical life changes, such as pregnancy, with potentially severe consequences for both the mother and pregnancy [4]. Despite this, many clinicians do not screen for EDs during pregnancy or feel equipped to provide sensitive care or to address the myriad complications should they have an ED [5, 6]. The purpose of this study was to evaluate a sensitivity training about eating disorders in pregnancy to provide greater knowledge and strategies on this topic for healthcare professionals so that they could combat biases and approach care with de-stigmatizing language and approaches.

### Eating disorders

The most common EDs that affects adult females are anorexia nervosa (AN), bulimia nervosa (BN), binge eating disorder (BED), and Other Specified Feeding and Eating Disorders (OSFED). AN is defined by a fear of gaining weight, body dysmorphia, and restricted eating. BN is characterized by persistent episodes of binge eating and a sense of lack of control, body dysmorphia, and compensatory behavior (through induced vomiting, periods of fasting, excessive exercise, and/or misuse of laxatives). BED also consists of recurrent episodes of binge eating; however, BED differs in that it is also associated with at least three of the following due to a sense of loss of control: eating more quickly than normal, overeating until uncomfortably full, eating large portions of food even when not hungry, and feeling guilt, shame, and/ or sadness after eating. OSFED can include AN, BN, or BED cases that present with symptomatology that do not meet frequency or duration criteria for diagnosis or have more varied presentations [1].

EDs can also be associated with complications throughout the body [7, 8]. Menstrual irregularity is a particularly important consideration for female patients with ED that negatively impacts endocrine and reproductive functions. Amenorrhea, or the absence of menstruation hypothesized to be the result of hypothalamic dysfunction, is common with individuals with AN and has been observed with individuals with OSFED and BN [9, 10]. Oligomenorrhea, which is defined as infrequent menstruation, has been observed in some patients with AN but is more common in patients with BN or OSFED [10]. BED is also associated with amenorrhea and oligomenorrhea in some patients [11]. Additional gynecological complications can arise in women with EDs as well, such as sexual dysfunction, polycystic ovarian syndrome (PCOS), and increased mortality due to gynecological cancers [10].

### Pregnancy and EDs

EDs are more common than previously understood during pregnancy, with best prevalence estimates at 5% [12, 13] and ranges from 0.6% to 27% [14–19], demonstrating there is a greater need to understand ED symptomatology during pregnancy [16]. Maternal EDs are associated with adverse pregnancy outcomes, including increased risk of hyperemesis and anemia during pregnancy and preterm birth, as well as neonatal outcomes such as giving birth to an offspring with microcephaly [20]. Other possible adverse neonatal outcomes include low birthweight, small size for gestational age, smaller head circumference [21] and perinatal death [12]. It is worth noting maternal and neonatal outcomes differ based on the type of ED the patient presents with [12], further indicating clinicians should understand ED symptomatology.

Women with AN have higher rates of unplanned pregnancy and abortion [22]. It is hypothesized absence of menstruation can mislead women to believe they are not fertile and to engage in risky behaviors, such as failure to use contraception [4]. Unplanned pregnancy can be particularly concerning because a woman may delay prenatal care or fail to acquire adequate nutrition for herself and the offspring. In addition, women with BN have more miscarriages, and women with AN are more likely to have Cesarean deliveries and give birth prematurely more often than the average woman [22, 23]. These findings did not differ for women with active vs. remitted AN, indicating a patient’s ED history can be especially important to their pregnancy and offspring’s health and demonstrating greater need for prenatal and perinatal care [23]. A study by Linna et al. [24] established that women with BED also have significantly high rates of miscarriage.

Although some studies have shown that pregnancy can lead to remission of some EDs, it can be a particularly vulnerable window for others; for example, individuals with BED seem to be particularly at risk [25]. Recurrence of ED may lead to higher incidence of postpartum depression [26]. One study found that for many women, ED symptoms improved during pregnancy but worsened postpartum [27]. A qualitative study by Tierney et al. [28] identified three types of women with ED during pregnancy: those who recovered during pregnancy and maintained that recovery postpartum, those who temporarily recovered during pregnancy and relapsed postnatally, and those who continued their ED behaviors throughout pregnancy. Although women with EDs can proceed through pregnancy differently, additional medical intervention, care, and monitoring is necessary for all.

### Current status of ED training for clinicians

In order to provide comprehensive medical care to women with ED, clinicians must be trained to properly diagnose, treat, and provide resources to patients with ED, yet numerous studies have indicated medical providers do not feel prepared to do so. Sixty-eight percent of clinician respondents from one study indicated they did not screen for EDs, and 59% of providers believed they did not have the skills to intervene with ED [29]. Similarly, 78% of clinicians reported having patients with EDs that they were unsure how to treat [6].

Mahr et al. [6] evaluated ED training in residency programs in internal medicine, pediatrics, family medicine, psychiatry, and child and adolescent psychiatry. The vast majority of programs across the United States did not offer any scheduled or elective rotations for ED, and of those that did, only a few were formal rotations. Child and adolescent psychiatry programs were found to offer the most clinical experiences with ED [6]. Psychiatry residents and resident physicians who had treated at least one patient with an ED were more comfortable treating patients with ED than those in non-psychiatry disciplines [30].

Although the majority of obstetrician and gynecologists (OB/GYNs) assess nutrition, body weight, BMI, and exercise, most do not assess ED history, body dysmorphia, binging, or purging [31]. Over 89% of OB/GYNs indicated they did not undergo adequate training for ED diagnosis or treatment during residency [31]. Considering the adverse neonatal and maternal outcomes that arise from maternal ED [20], it is critical that clinicians involved with treating women during pregnancy are trained to understand complications that may arise from ED. One possible solution is to develop training programs about ED for health professionals to improve their level of knowledge and confidence with treating ED [32].

### Proposed ED training

Based on the lack of medical training regarding ED and the importance of ED history during pregnancy, there is a need for additional training for clinicians surrounding ED symptomatology, complications, and implementation strategies. This study outlines a pilot test of a sensitivity training for clinicians with the goal of providing clinicians with additional training on ED to help clinicians recognize the importance of ED diagnosis for the treatment of patients in preconception (prior to pregnancy), prenatal (from conception to birth), and perinatal (time from becoming pregnant to one year post birth) periods and improve clinicians’ comfort and confidence in their ability to provide treatment to patients with ED. Sensitivitiy trainings are often conducted to provide information and increase awareness about a specific topic to allow for better care or interactions. This sensitivity training was designed to provide information of and awareness about eating disorders in pregnancy, address areas where clinicians may have biases, and offer strategies about more patient-centered care with de-stigmatizing language (person-first language) and practices (reduced stress options for weighing). In order to pilot test this training, we conducted qualitative and quantitative assessment of health professional survey responses prior to and after the sensitivity training and compared responses to those who were only provided a clinician reference document. Our primary aim was to determine if clinicians would feel more confident treating individuals with EDs during pregnancy after a short sensitivity training compared to a reference document. We hypothesized that after the sensitivity training, clinicians would feel more confident in implementing strategies and comfortable in their ability to treat ED patients than those only provided with a reference document. Our second aim was to determine if healthcare professionals would also endorse learning and behavior changes related to the sensitivity training compared to the reference document. We hypothesized that the sensitivity training would result in more substantial changes in participants’ perceptions of eating disorders’ relevance to overall treatment, comfort in providing resources, frequency of ability to introduce strategies, and interest in additional strategies/recommendations in treating patients with eating disorders.

## Methods

### Sensitivity training description

The sensitivity training consisted of an approximately 45-min PowerPoint (either delivered in person or voice-recorded over PowerPoint). It was broken into four different sections including: 1) an overall background on eating disorders, their prevalence, and treatment; 2) eating disorders in pregnancy including prevalence and lived experience through qualitative research; 3) observing example clinical encounters; and 4) recommendations for healthcare professionals. The example clinical encounters were videos that were scripted and recorded by some of the research team based on qualitative and quantitative research on this topic. One recorded video showed a less-than-ideal clinical encounter, which was followed by time for discussion or pause for thought to determine issues with the interaction. This was followed by a second video that corrected many of those issues and highlighted the differences between the two videos. Another key piece of the sensitivity training was a word comparison table that provided alternative terms for individuals to consider using in order to change the discourse with patients, such as using person-first language “individuals with anorexia nervosa” rather than “anorexic” or discussing “treatment seeking” rather than “struggling [with an eating disorder”. These recommendations were compiled from different sources to assist healthcare professionals with this population [33–37]. At the beginning and end of the sensitivity training, a QR code and link were included in the slide deck for the pre- and post-surveys.

### Reference document description

The reference document was developed based from the main areas highlighted in the sensitivity training: information about general eating disorders, pregnancy and eating disorders, consequences of eating disorders, and pertinent statistics and qualitative findings. The primary differences were that there was not attention provided to sensitivity and how to talk to individuals with eating disorders, nor were there example videos of what to do and what not to do. The reference document although providing extensive information, was considerably less interactive than the sensitivity training (see Appendix A for Reference Document).

### Data collection

Sampling was conducted using both convenience and snowball sampling in both in-person and virtual settings. One sensitivity training was conducted in person prior to the COVID-19 pandemic in January 2020 with a group of medical students in a large mid-Atlantic university’s medical school. It took place in a discussion-style classroom and was delivered to a nutrition-focused medical student group by E.C., Z.K., and C.D. Due to safety considerations during the COVID-19 pandemic, the training was recorded to be administered virtually via Grand Rounds and was sent to medical students at that same school as well as early professional listservs to complete study recruitment. The Grand Rounds comprised of medical professionals in Obstetrics and Gynecology which completed the training synchronously. Those who received the sensitivity training via medical school or professional listserv completed the virtual sensitivity training asynchronously. Virtual data collection ended in November 2021.

The clinician reference document survey was also disseminated virtually (due to continued COVID-19 precautions and ease of survey completion) between June 2021 and August 2021 via Survey Circle (an online survey collection platform), with restrictions of eligibility to those 18 years and older and who were healthcare professionals with clinical experience. Qualtrics software (Qualtrics, Provo, UT) was used to host and distribute the survey and no protected health information (PHI) was obtained.

This study was filed with West Virginia University’s Institutional Review Board and approved (IRB#: 1909705198).

### Trainer & data collector descriptions

The primary trainer for the sensitivity training was the principal investigator, E.C., who is a cis-gender White woman with a PhD in Social & Behavioral Sciences currently acting as an Assistant Professor. At the time of training, she had 10 + years of experience in public health, research, and eating disorders and a strong focus in eating disorders and pregnancy with a personal history of a past eating disorder. Pre-recorded virtual trainings and synchronous virtual trainings were also delivered by E.C. Assisting in the in-person sensitivity training were Z.K. and C.D. Z.K. is a cis-gender South Asian woman who is currently a M.D. candidate and has a BA in Biology. At the time of the training, she was an undergraduate student pursuing her bachelor’s degree. C.D. is a cis-gender White woman who is a PhD candidate in Social & Behavioral Sciences currently working full-time as a Research Specialist. At the time of the training, she was a Ph.D. student in Social & Behavioral Sciences with a Masters in Psychology and 5 + years of research experience. The goals of the research were explained to all participants in a cover letter, but a relationship was not established prior to trainings due to the virtual nature of some of the trainings and since the qualitative data collected was through open-ended survey responses rather than interviews or focus groups.

### Participants

Eligibility for both cohorts included being over 18 years of age and acting as healthcare professionals with clinical experience.

#### Clinician sensitivity training

A total of 55 consented to participate in the pre-survey once matched from pre-to post survey completion based on participant ID, it reduced the sample to 54.

#### Reference document

Seventy-five participants consented to participate. Ten consented but completed no more of the study, reducing the total to 65. Three completed the consent and demographics portion but did not complete the outcomes, reducing the sample further to 61 participants.

### Measures

#### Outcome measures

Outcomes were assessed through pre and post surveys with both quantitative and qualitative (open-ended responses) items. Questions were developed by adapting some of the Physician Attitudes and Knowledge Survey questions to be applicable to eating disorders in pregnancy [30]. Other questions were added and adapted that are typically used in assessing change (including knowledge, attidudes, and practice changes) from and needs for trainings in this area [29, 32].

##### Relevance of ED

Participants were asked: “If a patient has/had an eating disorder, how relevant do you feel it would be to their overall treatment?” Measured with a 5-point Likert-type response scale: Extremely relevant, Somewhat relevant, Neither relevant nor irrelevant, Somewhat irrelevant, and Extremely irrelevant.

##### Comfort providing resources

“How comfortable do you feel with providing patients with eating disorders with additional counseling/resources?” Measured by a 5-point Likert-type response scale: Extremely comfortable, Somewhat comfortable, Neither comfortable nor uncomfortable, Somewhat uncomfortable, and Extremely uncomfortable.

##### Implementing strategies

“How often do you feel you could implement alternative treatment strategies/recommendations in your daily practice?” Measured by a 5-point Likert-type response scale: Always, Often, Sometimes, Rarely, and Never.

##### Interest in additional strategies

“How interested would you be in implementing additional strategies/ recommendations meant for treating patients with eating disorders?” Measured by a 5-point Likert-type response scale: Extremely interested, Somewhat interested, Neither interested nor uninterested, Somewhat uninterested, and Extremely uninterested.

#### Clinician reference document survey

The clinician reference document combined the pre and post questions into one survey. There were six demographic questions, five pre-reference document questions, and five post-reference document questions in addition to two short answer questions. The reference document also ensured that participants spent at least 150 s (2.5 min) reading the reference document, which was enforced by a timed advance on Qualtrics (Qualtrics, Provo, UT). Immediately following the clinician reference document, participants had to answer three knowledge check questions about the document. These were: Q1: Of the following, which is false. “Pregnant women with eating disorders …” (answers: May report a decrease in concerns about body shape and weight due to their view of pregnancy being an acceptable reason to gain weight and have a larger body shape; should be considered a normal-risk pregnancy even though they are more likely to undergo Caesarian sections and have postpartum depression; may be more concerned with gestational weight; could have difficulty achieving nutritional needs for the fetus; are more likely to induce vomiting, misuse laxatives, and practice excessive exercise than those without an eating disorder); Q2: “Which of the following pairs of eating disorders and characteristics do not matchup?” (answers: 1. Anorexia nervosa– restricting food intake due to fear of gaining weight and/ or body dysmorphia that can result in significantly lower weight compared to the individual's minimal weight, 2. Bulimia nervosa– a disturbance in eating/ feeding from a lack of interest in or avoidance of food; significant nutritional deficit; dependence on nutritional supplements or feeding assistance; weight loss or failure to gain expected weight, 3. Other specified eating feeding and eating disorders– can include anorexia nervosa, bulimia nervosa, or binge eating disorder that do not present with typical criteria or symptoms, as well as purging disorder and night eating disorder, 4. Binge eating disorders– reoccurring episodes of binge eating associated with three of the following: feeling depressed or guilty after eating, eating large portions of food when not hungry, eating faster than normal, and eating until uncomfortably full.); and Q3: “Which of the following are health effects of eating disorders?” (answers: depression; electrolyte imbalance; tachycardia or bradycardia; anxiety; social withdrawal; osteoporosis; gastrointestinal complications, such as gastroesophageal reflux disease or gastric rupture; or all of the above). The correct answer is bolded above for each of the questions.

### Data analysis

#### Quantitative

Descriptive statistics were run on the full sample as well as separately for the sensitivity training and reference document. Differences on categorical variables between samples were analyzed using Fisher’s Exact tests due to small cell sizes. Mann–Whitney Wilcoxon (M-W W) tests were run on the difference (post–pre) scores of four primary outcomes to test equality in the two independent samples. This non-parametric approach was selected due to the ordinal nature of the outcomes. Additionally to understand how outcomes differed by key demographic characteristics that differed between the training and reference document group, sensitivity analyses were conducted stratifying results by sex and current student status. All quantitative analyses were conducted using SAS 9.4.

#### Qualitative

The qualitative data was approached using qualitative description, a data-near approach that involves less interpretation and keeps the data closer to the original in language and description [38]. Analysis was conducted of the two open-ended questions (Q1: “What additional training or resources do you think you need in order to treat patients that may have an eating disorder?” And Q2: “Do you utilize any particular methods or strategies when treating or interacting with a patient with an eating disorder?” and gleaning both major and subthemes from the written responses on two questions from both the sensitivity training and reference document [39]. Z.K. and E.C went through the steps of thematic analysis including familiarizing themselves with the data, creating initial codes inductively after reviewing responses several times, searching for themes, reviewing themes, and definining themes. Additionally, Z.K. and E.C. came to agreement on codes and discussed any discrepancies in coding. After coding, similar codes were collapsed into major and subthemes based on relevance. Data saturation was assured by no new themes arising in the responses after iterative review and coding as well as consensus between Z.K. and E.C. NVIVO 11 was used for data management and coding. Salient quotes were chosen through consensus between Z.K. and EC that were representative of each of these major and subthemes. Salient quotes were checked and verified for relevance to themes and the paper by C.D. and C.L.

### Applying the kirkpatrick model to results

The Kirkpatrick Model of Program Evaluation is an established approach to structure the evaluation of trainings and educational programs [40]. It has four levels of evaluation that can be applied to these programs including: Level 1: Reaction – how favorably participants respond to an intervention or training; Level 2: Learning – the degree to which participants gained knowledge and/or skills related to the main training outcomes; Level 3: Behavior – how participants changed their behavior based on the training; Level 4: Results – the return on expectations or whether training goals were achieved.

## Results

### Descriptive statistics

In the sensitivity training sample (*n* = 54), the majority of participants were medical students (81.1%), female (83%), ages 18–24 (50.9%), and white (81.5%). Although many had not chosen their specialty yet, the largest proportion with an identified specialty were in obstetrics and gynecology (*n* = 10; 30%). For the reference document sample (*n* = 61), the majority were professionals (72.1%) rather than students, female (63.9%), between the ages of 18–24 (42.6%), and White (62.3%). Additionally, 18.3% (*n* = 11) of participants had obtained a high school degree (or equivalent), completed some college, or held an associate’s degree. These individuals were working in the medical field but would have differing levels of care for patient.Characteristics for the full sample and by intervention group are listed in Tables 1 and 2.

**Table 1:** Descriptive statistics for demographics and student questions, for the full sample (*n* = 115) and separated by intervention group

Demographic	**Full sample (*n* = 115)**			**Sensitivity training (*n* = 54)**		**Reference document only (*n* = 61)**		Fisher’s exact *p*-value
	N	%	Recoded	N	%	N	%	
**Student**			**Student**					< 0.0001
Yes	60	52.6	Yes	43	81.1	17	27.9	
No	54	47.4	No	10	18.9	44	72.1	
**Education**			**Education**					< 0.0001
HS or equivalent	1	0.9	Coll/Assoc^b^	1	1.9	10	16.4	
Some college	7	6.1	Bachelor	39	73.6	20	32.8	
Associate	3	2.6	Post-grad	13	24.5	31	50.8	
Bachelor	59	51.8						
Master	27	23.7						
Professional	6	5.3						
Doctorate	11	9.7						
**Gender**								0.03
Male	31	27.2		9	17.0	22	36.2	
Female	83	72.8		44	83.0	39	63.9	
**Age category**			**Age**					0.23
18–24	53	46.5	18–24	27	50.9	26	42.6	
25–34	43	37.7	25–34	21	39.6	22	36.1	
35–44	12	10.5	35 +	5	9.4	13	21.3	
45–54	4	3.5						
55–64	1	0.9						
65 +	1	0.9						
**Race/Ethnic**			**Race/Ethnic**					0.03
American Indian	2	1.8	R&E min.^a^	10	18.5	23	37.7	
Asian	16	14.0	White	44	81.5	38	62.3	
Black/AA	3	2.6						
Hispanic	4	3.5						
White	80	70.2						
Two or more	7	6.1						
Other	2	1.8						

^a^racial & ethnic minority

^b^Part college or associates degree

**Table 2:** Descriptives

**Questions**	**Full sample (*n* = 115)**		**Sensitivity training (*n* = 54)**		**Reference document only (*n* = 61)**		**Mann–Whitney *p*-value**
	N/M SD		N/M SD		N/M SD		
1. **Relevance**: If a patient has/had an eating disorder, how relevant do you feel it would be to their overall treatment?							
Pre	1.5	0.9	1.2	0.6	1.7	1.0	0.005
Extremely relevant	77	67.5	44	83.0	33	54.1	
Somewhat relevant	28	24.6	8	15.1	20	32.8	
Neither	3	2.6	0	0	3	4.9	
Somewhat irr	3	2.6	0	0	3	4.9	
Extremely irr	3	2.6	1	1.9	2	3.3	
Post	1.5	0.9	1.0	0.2	1.9	1.1	< 0.0001
Extremely relevant	71	73.2	43	97.7	28	52.8	
Somewhat relevant	14	14.4	1	2.3	13	24.5	
Neither	6	6.2	0	0	6	11.3	
Somewhat irr	4	4.1	0	0	4	7.6	
Extremely irr	2	2.1	0	0	2	3.8	
2. **Resources**: How comfortable do you feel with providing patients with eating disorders with additional counseling/resources?							
Pre	2.4	1.0	2.9	1.0	2.1	0.8	0.0002
Extremely comfort	17	14.9	4	7.6	13	21.3	
Somewhat comfort	51	44.7	16	30.2	35	57.4	
Neither	28	24.6	19	35.9	9	14.8	
Somewhat uncomf	15	13.2	12	22.6	3	4.9	
Extremely uncomf	3	2.6	2	3.8	1	1.6	
Post	2.2	0.9	2.1	0.6	2.3	1.1	0.07
Extremely comfort	17	17.5	5	11.4	12	22.6	
Somewhat comfort	58	59.8	33	75.0	25	47.2	
Neither	12	12.4	4	9.1	8	15.1	
Somewhat uncomf	8	8.3	2	4.6	6	11.3	
Extremely uncomf	2	2.1	0	0	2	3.8	
3. **Family**: If a patient’s family members were to be involved, how comfortable would you feel providing them with accurate, helpful information?							
Pre	2.4	1.1	2.9	1.1	2.0	0.9	0.0004
Extremely comfort	19	16.7	4	7.6	15	24.6	
Somewhat comfort	53	46.5	19	35.9	34	55.7	
Neither	21	18.4	13	24.5	8	13.1	
Somewhat uncomf	17	14.9	14	26.4	3	4.9	
Extremely uncomf	4	3.5	3	5.7	1	1.6	
4. **Strategies**: How often do you feel you could implement alternative treatment strategies/ recommendations in your daily practice?							
Pre	2.6	1.0	2.5	0.9	2.6	1.0	0.27
Always	15	13.2	8	15.1	7	11.5	
Often	42	36.8	18	34.0	24	39.3	
Sometimes	41	36.0	23	43.4	18	29.5	
Rarely	10	8.8	2	3.8	8	13.1	
Never	6	5.3	2	3.8	4	6.7	
Post	2.3	1.0	1.9	0.8	2.6	1.0	0.002
Always	23	23.7	15	34.1	8	15.1	
Often	35	36.1	19	43.2	16	30.2	
Sometimes	28	28.9	10	22.7	18	34.0	
Rarely	10	10.3	0	0	10	18.9	
Never	1	1.0	0	0	1	1.9	
5. **Additional Strategies:** How interested would you be in implementing additional strategies/ recommendations meant for treating patients with eating disorders?							
Pre	1.6	0.8	1.3	0.5	1.9	0.92	< 0.0001
Extremely int	61	53.5	40	75.5	21	34.4	
Somewhat int	41	36.0	11	20.8	30	49.2	
Neither	7	6.1	2	3.8	5	8.2	
Somewhat unint	4	3.5	0	0	4	6.6	
Extremely unint	1	0.9	0	0	1	1.6	
Post	1.6	1.0	1.1	0.3	2.1	1.1	< 0.0001
Extremely int	59	60.8	41	93.2	18	34.0	
Somewhat int	23	23.7	3	6.8	20	37.7	
Neither	8	8.3	0	0	8	15.1	
Somewhat unint	6	6.2	0	0	6	11.3	
Extremely unint	1	1.0	0	0	1	1.9	
6. **Frequency**: How frequently (that you are aware of) have you treated a patient with an eating disorder?							
Post	3.6	1.2	3.8	1.1	3.3	1.2	0.15
Frequently	2	2.1	1	2.3	1	1.9	
Somewhat freq	21	21.7	5	11.4	16	30.2	
Neither	22	22.7	10	22.7	12	22.6	
Somewhat infreq	25	25.8	12	27.3	13	24.5	
Infrequently	27	27.8	16	36.4	11	20.8	

There were significant differences across the groups for student, with more students in the sensitivity training group (*N* = 43; 81.1% vs. *N* = 17; 27.9%); gender, with more females in the sensitivity training group; and racial and ethnic identity, with a larger proportion of White participants in the sensitivity training (*N* = 44, 81.5% vs. *N* = 38, 62.3%).

To help better evaluate the sensitivity training, qualitative and quantitative outcomes are organized according to the corresponding levels of the Kirkpatrick model [40]. This provides a greater degree of evidence for each level and the training overall.

**Fig. 1:**
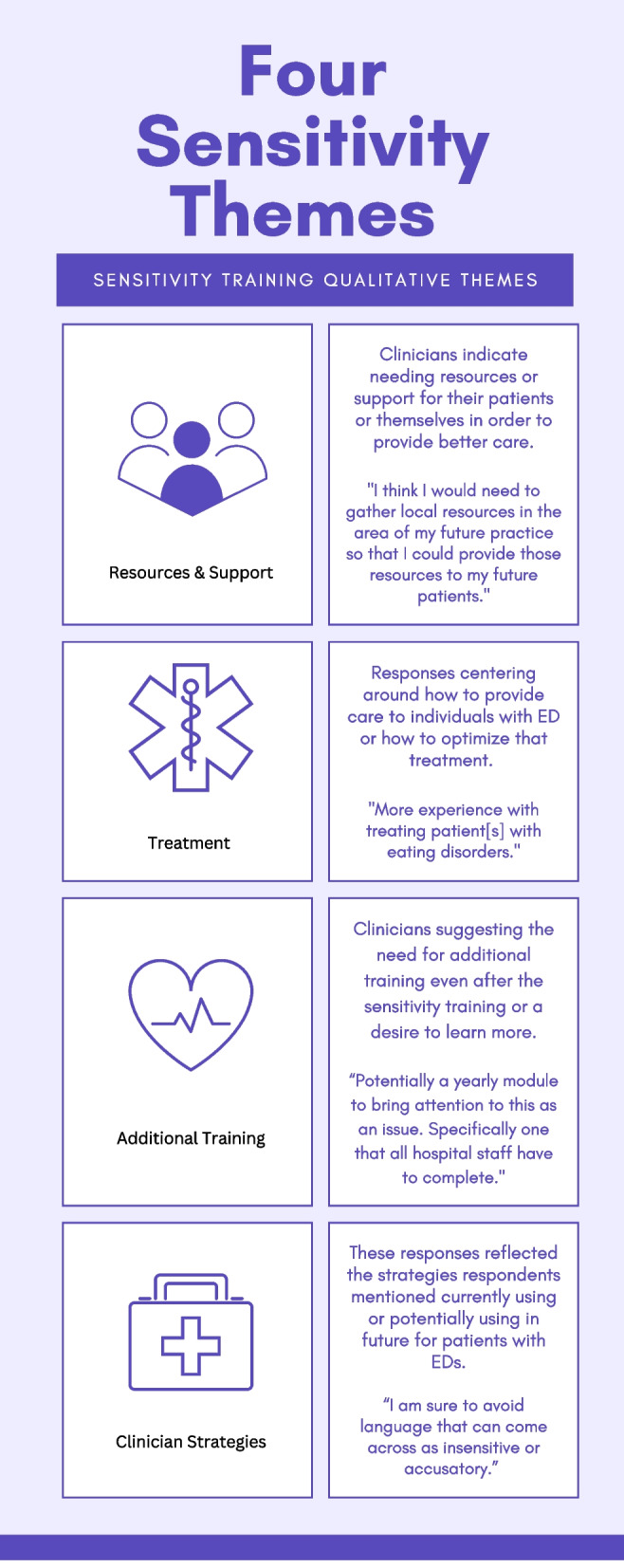
Four qualitative sensitivity training themes

The thematic analysis of open-ended responses showed four major themes that emerged from open-ended responses from both the sensitivity training and the reference document condition. The two open-ended questions asked: 1) Which additional training or resources do you think you need in order to treat patients that may have an eating disorder? And 2) Do you utilize any particular methods or strategies when treating or interacting with a patient with an eating disorder? See Fig. 1 for a summary of the four main themes with descriptions.

### Level 1: reaction

Although questions were not asked specifically about their reaction to the training or reference document, 8 mentioned specific aspects of the training or document in the open-ended questions and how they found them positive or favorable. All eight of these were within the sensitivity training group and included comments such as “this lecture was very helpful in presenting strategies (participant 95)” and the “sensitivity training helps with using words that respect the patient (participant 71)”.

### Level 2: learning

A majority of individuals completed each of the knowledge check questions correctly: knowledge check 1 (32, 57.1%); knowledge check 2 (37, 66.1%); and knowledge check 3 (43, 76.8%). Based on the knowledge they gained from the training, participants showed an increased interest in additional strategies/recommendations in treating patients with eating disorders compared to the reference document group (M = 0.14 ± 0.41 vs. M = -0.23 ± 1.07; M-W W *p* = 0.009). See Table 3 for full details on differences between questions in the two groups.

**Table 3:** Differences over time by group; negative scores indicate decreases in variables and positive scores indicate increases

Differences score	Sensitivity training (*n* = 43)		Reference document only (*n* = 53)		Mann–Whitney Wilcoxon *p*-value
	M (SD)	Median (min, max)	M (SD)	Median (min, max)	
Relevance^a^	0.23 (0.68)	0 (0, 4)	-0.15 (1.05)	0 (-3, 3)	0.018
Resources^b^	0.79 (0.86)	1 (-1, 3)	-0.19 (1.09)	0 (-4, 3)	< 0.0001
Strategies^c^	0.49 (0.70)	0 (0, 2)	-0.06 (1.06)	0 (-3, 3)	0.001
Additional Strategies^d^	0.14 (0.41)	0 (-1, 1)	-0.23 (1.07)	0 (-3, 3)	0.009

^a^ Relevance of ED to treatment

^b^ Comfort providing patients with resources

^c^ Frequency of implementing strategies

^d^ Interest in implementing additional strategies

A theme around *Resources & Support* comprised responses that indicated needing either resources and or support for themselves or patients so that they could better provide care. Participants indicated the need for “specialist training and [a] list of support networks [reference document (RD) participant 42]” and a “list of resources, more information on how to recognize undiagnosed eating disorders [sensitivity training (ST) participant 64]” illustrating the desire for easy documents to turn to. Others also stated the importance of compiling local resources for patients: “information about local resources for patients – specialists, support groups ST participant 64”; “I think I would need to gather local resources in the area of my future practice so that I could provide those resources to my future patients. ST participant 108” There was an overall expression of additional support needed for physicians so that they could provide these resources to patients: “Becoming educated about holistic care for these patients and what outside resources are available. ST participant 76”.

Participants also highlighted a theme about *Additional Training* indicating that participants were interested in the topic and felt they gained useful information from either the training or reference document, but still wanted additional training to be able to learn more. They cited wanting: “training workshops about specific types of eating disorders and how to best advise patients accordingly (RD participant 54)” as well as “knowledge on how eating disorders evolve and relapse, mindfulness and intuitive eating knowledge, ACT [Acceptance and Commitment Therapy], EMDR [Eye movement desensitization and reprocessing], relaxation (RD participant 5)”. Some indicated the need for these kinds of trainings to be regularly implemented to ensure knowledge retention: “Potentially a yearly module to bring attention to this as an issue. Specifically one that all hospital staff have to complete. (ST participant 63)” Having a pragmatic tool to use was also important to participants, indicated by them stating wanting training in “how to screen more quickly in patients. (RD participant 58)”.

### Level 3: behavior

Both quantitative and qualitative data can provide evidence and context on behavior change as a result of the training. Compared with the reference document group, the frequency of participant’s ability to introduce strategies to help those with EDs increased (M = 0.49 ± 0.68 vs. M = -0.06 ± 1.06; M-W W *p* = 0.001). Participants also reported experiencing more comfort in providing resources post sensitivity training compared to the reference document (M = 0.79 ± 0.86 vs. M = -0.19 ± 1.09; M-W W *p* < 0.0001).

Additionally, a theme on clinical strategies spoke to areas of behavior change related to the training. The theme on Clinician Strategies illustrated strategies clinicians currently or would employ with patients with EDs. Some individuals mentioned the skills and strategies they learned through the sensitivity training, especially around the importance of language, stating the importance of “being sensitive to my language and responses about weight and eating (ST participant 76)”. Some already employed those techniques, responding that “I am sure to avoid language that can come across as insensitive or accusatory (ST participant 63).” Participants also discussed ways to encourage patients to help change ED behaviors: “I would also be sure to mention the negative implications of how an eating disorder could affect their conditions and treatment strategies, and be sure they understand this” (ST participant 63). A person-centered approach was also mentioned as critical, with participants stating even after the reference document they would “use more tailored strategies related to eating disorders, probably based on psychological evidence (RD participant 54)”.

### Level 4: results

The sensitivity training compared to the reference document group was associated with increases to participants’ perception of eating disorders’ relevance to overall treatment (M = 0.23 ± 0.68 vs. M = -0.15 ± 1.05; M-W W *p* = 0.018). This indicates a greater understanding of EDs and how they may influence perinatal maternal and child health outcomes, which was one of the main goals of the training.

Qualitatively, the treatment theme focused on participant responses around how to provide treatment to individuals with EDs or what was needed to optimize treatment. Participants stated they needed “a good risk assessment and treatment guidance (RD participant 26)” as well as “more experience with treating patient[s] with eating disorders (ST participant 69)”. Others also focused on the importance of providing “timely treatment (RD participant 23)” for individuals with EDs based on the training they received. This illustrates a need to treat EDs in pregnancy differently and a commitment to do so.

### Sensitivitiy analyses

Due to some differences in the demographic characteristics between the training and reference document groups, we conducted sensitivity analyses on the main outcomes based on student status and sex. These findings showed significant increases in the sensitivity training for comfort in providing resources for men (M = 0.57 ± 0.79 vs. M = -0.57 ± 1.50; M-W W *p* = 0.03) and significant increases for women in resources (M = 0.83 ± 0.88 vs. M = 0.06 ± 0.62; M-W W *p* = 0.0002) and ability to introduce strategies (M = 0.50 ± 0.70 vs. M = -0.13 ± 0.83; M-W W *p* = 0.0009) compared with the reference document group. See Supplementary Table 1 for full sensitivity analyses results by sex.

For student status, the analyses were associated with students’ perception of eating disorders’ relevance to overall treatment (M = 0.25 ± 0.73 vs. M = -0.33 ± 1.29; M-W W *p* = 0.006) and comfort in providing resources (M = 0.86 ± 0.90 vs. M = 0.00 ± 1.07; M-W W *p* = 0.0003) compared with the reference document group. Non-student status had no significant differences. See Supplementary Table 2 for full sensitivity analyses results by student status.

## Discussion

The purpose of this study was to pilot test a sensitivity training geared toward clinicians who are providing treatment for patients with an ED diagnosis throughout their preconception, prenatal, and perinatal stages while improving comfort and confidence in clinicians’ ability to provide treatment to this population. The results from this pilot study support our hypothesis that clinicians would feel more confidence in implementing strategies and comfort in their ability to treat patients with an ED. While both the sensitivity training and the reference document increased clinicians’ comfort and confidence, the results show that the training was more effective. Going forward the reference document could be used in conjunction with the sensitivity training to reinforce information provided.

When understood within the Kirkpatrick model of program evaluation, we have all four levels of evidence illustrated in our evaluation of the sensitivity training. These levels of evidence show positive reactions to the sensitivity training compared with the reference document, improvements in learning and knowledge post training, behavior changes to be implemented, and that health professionals believed that EDs were relevant to care indicating a positive change in return on expectations. The results support our hypothesis that the sensitivity training would result in more significant changes in participants’ perceptions of eating disorders’ relevance to overall treatment, comfort in providing resources, frequency of ability to introduce strategies, and interest in additional strategies/recommendations in treating patients with eating disorders in comparison to the reference document group. These significant differences suggest that the sensitivity training provides greater improvements in the intended training outcomes than the reference document. This is likely due to the in-depth nature of the training compared to the reference document.

The thematic analysis of open-ended responses from both groups produced four major themes covering areas in which clinicians desire more information as well as strategies they already utilize. The first theme, *Resources and support*, showed that participants would like easily accessible information on resources they can suggest to their patients. The second theme, *Treatment,* found that clinicians recognize a general need for more experience treating patients with ED, good risk assessment, treatment guidance, and the importance of timely treatment. In the next theme, *Additional training*, participants were more interested in the subject, noting the usefulness of the training but also wanting more specified training (AN, BED, OSFED, etc.) in treating and recognizing individual EDs. There was also an expressed need for a more time efficient ED screening tool valid during pregnancy. Since this sensitivity training was piloted, Claydon and colleagues [41] have developed a rapid screening tool to identify eating disorders during pregnancy named the Prenatal Eating behaviors screening tool (PEBS). The PEBS tool consists of 12 items capable of reliably detecting eating disorders throughout all three trimesters. The final theme, *Clinician Strategies*, revealed that clinicians want to be able to effectively communicate with patients about EDs and have a desire for person-centered approached to treatment. It was also noted that some clinicians were generally aware of the language they use around food and weight prior to participating in this sensitivity training.

The qualitative findings also align with the existing literature, highlighting that clinicians do not feel prepared to diagnose, treat or provide resources to patients with ED without proper training [5, 29]. However, the quantitative findings from this short training resulted in a notable increase in clinicians’ understanding of EDs which can provide context and recommendations for future clinical trainings and continuing education courses. For example, many participants indicated a need for additional training and practice-based learning in the qualitative responses, paving the way for the possibility of developing and implementing a continuing medical education (CME) course on this topic.

Additionally, with our stratified sensitivity analysis, we found that students had significant findings, but non-students did not. This could be due to the fact that there were more students within the sensitivity training group, whereas the reference document group was predominantly more new professionals or professionals. Another explanation could be that students are embedded in a context of learning and may be more likely to change their ideas and attitudes rather than busy professionals. The difference found with more changes among women than men could be due to similar group differences of fewer men in the sensitivity training. More research with larger samples will need to be conducted to tease out why there are some differences among differing student status and sex.

Due to the risks and unique challenges of eating disorders during pregnancy and the fact that the majority of clinician respondents not feeling prepared or certain of how to screen or treat these individuals, there is a clear need for training in this area [20, 21, 27, 28]. Our findings illustrate that this type of training, including an aspect on sensitivity, can be effectively delivered to start the process of further education for clinicians can help to improve their knowledge and confidence treating patients with ED [32].

### Limitations

The sample size of this study is relatively small; however, we were still able to find some compelling results. The ongoing pandemic changed the way our training was disseminated, and we cannot be sure whether the virtual trainings slightly attenuated the outcomes. However, other evidence suggests that other trainings for medical students, including rotations, have been effectively conducted virtually with positive results [42]. Sampling was done through convenience and snowball sampling, rather than through a randomized method, which resulted in groups that differed in many characteristics. These differences were exacerbated by the pandemic which necessitated other ways to collect data rather than the previously planned in-person trainings of medical students. Future randomized studies will be conducted with these trainings to increase the evidence base. Additionally, the questionnaire utilized for the quantitative pre-post responses was not validated. However, it was adapted from the Physician Attitudes and Knowledge Survey which is designed for physician attitudes and knowledge around EDs [30]. One other limitation is that due to small sample sizes among different racial and ethnic identities, we had to collapse our categories into White and racial and ethnic minorities. This does diminish our ability to understand differences between racial and ethnic groups, but that was not possible due to the smaller sample sizes. Future studies will recruit larger groups, oversampling for racial and ethnic minorities to allow for a better distinction of these important aspects.

### Strengths

However, there are several strengths of this study including the ability to compare a longer sensitivity training to a reference document to understand the additional contributions of that in-depth training. The knowledge checks imbedded in the reference document also give us an indication of the level of knowledge individuals gained from the reference document. Additionally, the qualitative results help to strengthen our findings by adding unique insight of future areas to develop more resources, training, and understand the strategies that are currently being used or could be used by clinicians to help patients with eating disorders during pregnancy. Although respondents were not contacted for member checking of responses, data triangulation with quantitative responses was used to ensure trustworthiness of the data. Additionally, for the in-person sensitivity training, debriefing occurred after the training with a question/answer/feedback session. This session raised some of the same feedback brought up in qualitative responses.

## Conclusion

Given that EDs can lead to significant medical consequences throughout the body especially during pregnancy [7, 8], medical professionals of all disciplines should be trained to treat ED. In order to better serve ED patients, clinicians need additional resources. Resources can include information about word choice or sensitivity, resources for ED patients, training modules, and practice-based learning. Future directions from this study include additional translation to ensure that clinicians have access to these needed resources and training.

### Supplementary Information

**Additional file 1. **Reference document synthesizing information about eating disorders and pregnancy provided to clinicians with pre and post survey.

**Additional file 2: Supplementary Table 1.** Stratification of outcomes by sex. **Supplementary Table 2.** Stratification of outcomes by student status.

## Data Availability

Data is available upon reasonable request from the authors. Please contact Dr. Elizabeth Claydon at Elizabeth.claydon@hsc.wvu.edu with requests.

## Abbreviations

ACT: Acceptance and Commitment Therapy
AN: Anorexia Nervosa
BN: Bulimia Nervosa
BED: Binge eating disorder
CME: Continuing Medial Education
EMDR: Eye movement desensitization and reprocessing
EDs: Eating disorders
M-W W: Mann–Whitney Wilcoxon
PEBS: Prenatal Eating behaviors screening tool
PHI: Protected health information
OB/GYNs: Obstetrician-gynecologists
OSFED: Other Specified Feeding and Eating Disorders
