# Supplementary material for: “It is more isolating to patients if you aren’t familiar with the resources”: a pilot test of a clinician sensitivity training on eating disorders in pregnancy
Source: BMC Med Educ. 2023 Dec 6;23:924. doi: 10.1186/s12909-023-04894-x (PMC10699011; doi:10.1186/s12909-023-04894-x)
Supplement: Supplementary file 1 — Additional file 1. Reference document synthesizing information about eating disorders and pregnancy provided to clinicians with pre and post survey. [file 12909_2023_4894_MOESM1_ESM.pdf]

HEALING BODIES HEALTHY BABIES

OVERVIEW OF EATING DISORDERS

- Eating disorders have the second highest mortality rate of any psychiatric disorder.
- Hospitalizations due to EDs increased by 18% from 1999-2006. The rate of hospitalizations of children under 12 has increased by 119%.

CHARACTERISTICS OF EATING DISORDERS

Based on DSM-5

ANOREXIA NERVOSA (AN)

Fear of gaining weight; body dysmorphia; food intake restrictions, leading to a significantly low weight compared to what is minimally for that individual. Least common but most fatal due to malnutrition.

BULIMIA NERVOSA (BN)

Persistent episodes of binge eating; persistent behaviors aimed to prevent weight gain (induced vomiting, fasting, excessive exercise, misuse of laxatives); body dysmorphia; guilt or shame after binge eating episodes.

BINGE EATING DISORDERS (BED)

Reoccurring episodes of binge eating which are associated with at least three of the following: eating faster than normal, eating until uncomfortably full, eating large portions of food when not hungry, feeling depressed or guilty after eating.

AVOIDANT RESTRICTIVE FOOD INTAKE DISORDER (ARFID)

An eating/feeding disturbance such as a lack of interest in food or an avoidance of food; weight loss or failure of expected weight gain; significant nutritional deficit; dependence on nutritional supplements or feeding assistance.

OTHER SPECIFIED FEEDING AND EATING DISORDERS (OSFED)

Could include atypical AN, BN or BED criteria or less frequent symptoms; purging disorder (reoccurring purging behavior with the goal to influence body weight or shape); night eating syndrome (reoccurring episodes of night eating)

HEALTH EFFECTS OF EATING DISORDERS

MENTAL

- Depression
- Anxiety
- Withdrawal from socializing
- Increased self-awareness

PHYSICAL

- Electrolyte imbalance
- Tachycardia
- Bradycardia
- Osteoporosis
- Gastroesphageal Reflux Disease (GERD)
- Gastric Rupture

EATING DISORDERS DURING PREGNANCY

- Of women that presented signs of oligomenorrhea or amenorrhea, 58-76% had clinical implications of EDs, but none had revealed symptoms to their providers.
- A reemergence of symptoms can occur postpartum due to the increased stress and pressure to lose pregnancy weight
- The risk of miscarriages and induced abortions is increased in patients with a history of EDs.
- Women who have AN or BN are more likely to have positive changes to their eating habits while pregnant, while women who have BED are more likely to have negative changes to their eating habits or experience a relapse.
- Over 5% of women experience a form of ED during pregnancy.
- Post pregnancy, women with EDs are more likely to have troubles breastfeeding than those without EDs.
- The risk of postnatal depression is increased in women with EDs.
- The prevalence of disordered eating during pregnancy ranges from 0.6% to 27.8%.
- Pregnancy experiences protect against body dissatisfaction
- Protective factors disappear postpartum
- Three types of women identified
  - Women recover during pregnancy & maintain after
  - Temporarily recovered during pregnancy
  - Continued ED behaviors during pregnancy

EATING DISORDERS & PERINATAL COMPLICATIONS

- The children of women with EDs are more likely to have EDs, diabetes mellitus, hypertension, and heart disease.
- EDs during pregnancy can lead to fetal malnutrition, an increased exposure to stress hormones, reduced birth weight, and labor difficulties.
- Consequently, the infants are at risk for behavioral issues, increased reactions to stress, and decreased cognitive abilities during development.
- Pregnant women with EDs:
  - Are more concerned with gestational weight gain than those without.
  - May have difficulty trying to fulfill nutritional needs for the fetus, while also coping with body weight and shape changes.
  - May report a decrease in concerns about body shape and weight due to their view of pregnancy being an acceptable reason to gain weight and have a larger body shape.
  - Are more likely to induce vomiting, misuse laxatives, and practice excessive exercise than those without EDs.
  - Should be considered a high-risk pregnancy due to the increased risk of cesarean sections and postpartum depression.

MAIN THEMES OF EATING DISORDERS AND PREGNANCY

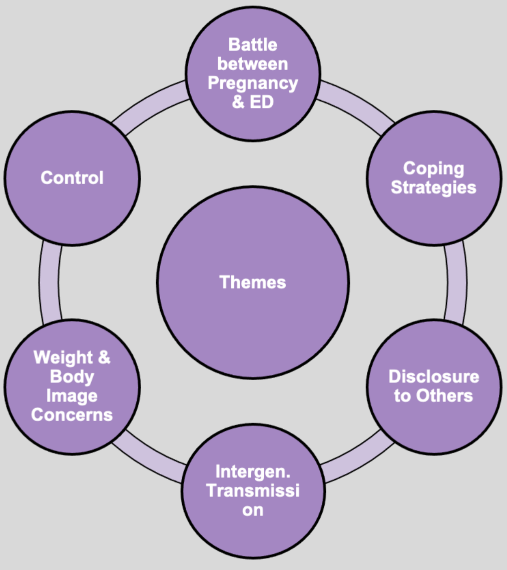

Themes derived from a qualitative study (Claydon et al.,2018)

American Psychiatric Association. (2013). Diagnostic and statistical manual of mental disorders : DSM-5 (5th ed.). American Psychiatric Association Publishing.

American Psychiatric Association. (2019). Pocket guide for the assessment and treatment of eating disorders. (J. Lock, Ed.) (First). American Psychiatric Association Publishing.

Bannatyne, A. J., McNeil, E., MacKenzie-Shalders, K., Stapleton, P., & Watt, B. (2019). Disordered eating measures validated in pregnancy samples: a systematic review. Eating Disorders, (2019). https://doi.org/10.1080/10640266.2019.1663478

Chan, C. Y., Lee, A. M., Koh, Y. W., Lam, S. K., Lee, C. P., Leung, K. Y., & Tang, C. S. K. (2019). Course, risk factors, and adverse outcomes of disordered eating in pregnancy. The International Journal of Eating Disorders, 52(6), 652–658. http://doi.org/10.1002/eat.23065

Pasalackis, G., Zwaan, M. (2019). Clinical management of females seeking fertility treatment and of pregnant females with eating disorders. European Eating Disorders Review, 27, 215-223. https://doi.org/ 10.1002/erv.2667

Zerwas, S., Claydon, E., & Barnes, Diana Lynn, 1818-887-1312, The Center for Postpartum Health (2014). Women's reproductive mental health across the lifespan. Eating disorders across the life-span: from menstruation to menopause (pp. 237–261). essay, Springer International Publishing. https://doi.org/10.1007/978-3-319-05116-1_13
