# Supplementary material for: “It is more isolating to patients if you aren’t familiar with the resources”: a pilot test of a clinician sensitivity training on eating disorders in pregnancy
Source: BMC Med Educ. 2023 Dec 6;23:924. doi: 10.1186/s12909-023-04894-x (PMC10699011; doi:10.1186/s12909-023-04894-x)
Supplement: Supplementary file 2 — Additional file 2: Supplementary Table 1. Stratification of outcomes by sex. Supplementary Table 2. Stratification of outcomes by student status. [file 12909_2023_4894_MOESM2_ESM.docx]

Supplementary Table 1. Stratification of outcomes by sex.

| **Sensitivity Training** | | | |  | **Reference Document** | | | | **Mann-Whitney p-value** |
| --- | --- | --- | --- | --- | --- | --- | --- | --- | --- |
| **Male** | | | | | | | | |  |
| **Variable** | **N** | **Mean** | **SD** |  | **Variable** | **N** | **Mean** | **SD** |  |
| Relevance | 7 | 0.14 | 0.38 |  | Relevance | 21 | -0.48 | 1.29 | 0.08 |
| Resources | 7 | 0.57 | 0.79 |  | Resources | 21 | -0.57 | 1.50 | 0.03* |
| Strategies | 7 | 0.43 | 0.79 |  | Strategies | 21 | 0.05 | 1.36 | 0.38 |
| Add. Info. | 7 | 0.29 | 0.49 |  | Add. Info. | 21 | -0.33 | 1.49 | 0.1 |
|  |  |  |  | **Female** |  |  |  |  |  |
| **Variable** | **N** | **Mean** | **SD** |  | **Variable** | **N** | **Mean** | **SD** |  |
| Relevance | 36 | 0.25 | 0.73 |  |  | 32 | 0.06 | 0.80 | 0.33 |
| Resources | 36 | 0.83 | 0.88 |  |  | 32 | 0.06 | 0.62 | 0.0002* |
| Strategies | 36 | 0.50 | 0.70 |  |  | 32 | -0.13 | 0.83 | 0.0009* |
| Add. Info. | 36 | 0.11 | 0.40 |  |  | 32 | -0.16 | 0.68 | 0.08 |

Analyses are run using delta scores.

Supplementary Table 2. Stratification of outcomes by student status.

| **Sensitivity Training** | | | |  | **Reference Document** | | | | **Mann-Whitney p-value** |
| --- | --- | --- | --- | --- | --- | --- | --- | --- | --- |
| **Not Student** | | | | | | | | |  |
| **Variable** | **N** | **Mean** | **SD** |  | **Variable** | **N** | **Mean** | **SD** |  |
| Relevance | 7 | 0.14 | 0.38 |  | Relevance | 38 | -0.08 | 0.94 | 0.47 |
| Resources | 7 | 0.43 | 0.53 |  | Resources | 38 | -0.26 | 1.11 | 0.08 |
| Strategies | 7 | 0.00 | 0.00 |  | Strategies | 38 | -0.21 | 1.09 | 0.51 |
| Add. Info. | 7 | 0.14 | 0.38 |  | Add. Info. | 38 | -0.39 | 1.03 | 0.08 |
| **Student** | | | | | | | | |  |
| **Variable** | **N** | **Mean** | **SD** |  | **Variable** | **N** | **Mean** | **SD** |  |
| Relevance | 36 | 0.25 | 0.73 |  | Relevance | 15 | -0.33 | 1.29 | 0.006* |
| Resources | 36 | 0.86 | 0.90 |  | Resources | 15 | 0.00 | 1.07 | 0.003* |
| Strategies | 36 | 0.58 | 0.73 |  | Strategies | 15 | 0.33 | 0.90 | 0.18 |
| Add. Info. | 36 | 0.14 | 0.42 |  | Add. Info. | 15 | 0.20 | 1.08 | 0.91 |

Analyses are run using delta scores.
